# 389. PediCoViral: Clinical Outcomes of COVID-related Co-viral infections in Children

**DOI:** 10.1093/ofid/ofad500.459

**Published:** 2023-11-27

**Authors:** Brenda I Anosike, Shiv M Sharma, Kiera Sarill, Jassour Alrikaby, Tim Q Duong, Betsy Herold

**Affiliations:** Children's Hospital at Montefiore, The Bronx, New York; Montefiore Medical Center/Albert Einstein College of Medicine, Bronx, New York; Children’s Hospital at Montefiore, Albert Einstein College of Medicine, Bronx, New York; Montefiore/Albert Einstein, 3411 Wayne Avenue, New York; Montefiore Medical Center/Albert Einstein College of Medicine, Bronx, New York; Albert Einstein College of Medicine, Bronx, New York

## Abstract

**Background:**

During the earlier phase of the COVID-19 pandemic, published reports demonstrated low rates of COVID co-viral infections. Upon relaxing public health measures, the epidemiology of known respiratory viral pathogens had dramatically shifted as the pandemic evolved. Little is known about the clinical impact of co-viral infections in children post-Omicron wave. This study investigates the clinical outcomes among children, adolescents, and young adults who test positive for SARS-CoV-2 [COVID] alone compared to those with COVID co-viral infection with either RSV or Influenza [Flu] A/B [denoted as COVID+]

**Methods:**

We conducted a retrospective study of subjects ≤ 21 years of age who tested positive for SAR-CoV-2 presenting to the Children’s Hospital at Montefiore, Bronx, NY from 12/1/2021 to 1/15/2023. Laboratory confirmation was established using a multi-target PCR assay for influenza A/B, RSV, and SARS-CoV-2. The primary outcomes were hospitalization, need for invasive mechanical ventilation (IMV), and mortality. Demographic and patient outcomes were extracted using ATLAS database.

**Results:**

Among 8307 subjects who tested positive for COVID alone, 8.5% (708) were hospitalized with a mean age of 8.7 years (SD=7.8) while mean age of those with COVID+ was 5.0 years (SD=4.4). Hospitalization rates for COVID alone, COVID+Flu, and COVID+RSV were 6.4%, 32.2%, and 57.7%, respectively (p< 0.001). COVID positive children ≤ 5 years of age with or without a second virus were more likely to be hospitalized than other age group (p< 0.001). When comparing COVID+Flu vs. Flu alone (32.2% vs. 4.3%, p< 0.001) or COVID+RSV vs. RSV alone (57.7% vs. 15.8%, p< 0.001), hospitalization rates were higher for those with COVID co-viral infections. No increase in mortality was observed.

**Figure 1.**
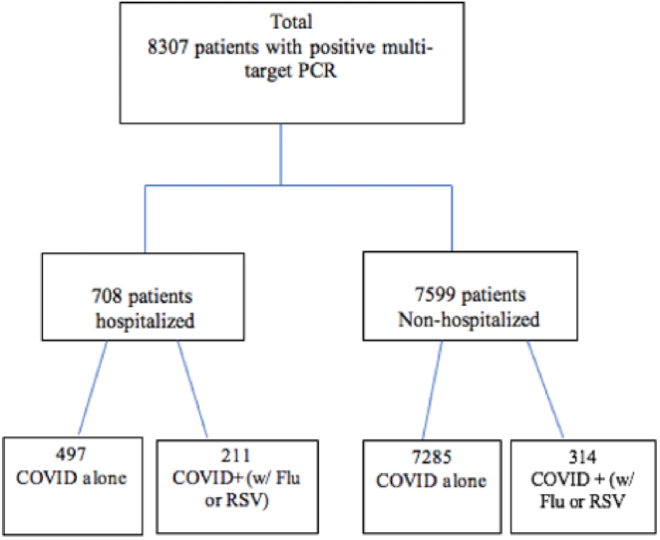
Patient Flow Chart

**Table 1.**
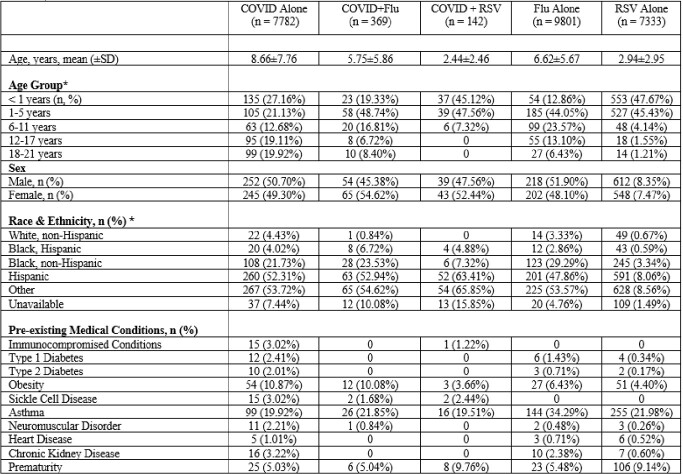
Patient Demographics and Characteristics *unadjusted

**Table 2.**
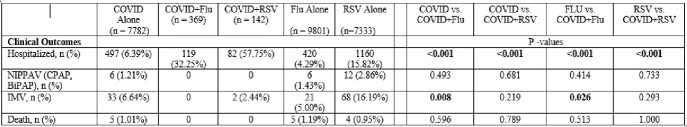
Clinical outcomes of pediatric patients

**Conclusion:**

This single center retrospective study demonstrates that SARS-CoV-2 coinfection with RSV or Influenza is associated with increased risk for hospitalization compared to SARS-CoV-2, RSV or influenza alone. Hospitalization rates were higher in younger children. Further studies are needed to identify the mechanisms driving the increased morbidity associated with these coinfections.

**Disclosures:**

**All Authors**: No reported disclosures

